# Cystitis in the Female—Case Illustrating Treatment1Read at the 36th annual meeting of the Medical Association of Central New York, held at Auburn, September 22, 1903.

**Published:** 1903-12

**Authors:** W. L. Wallace

**Affiliations:** Syracuse


### Cystitis in the Female—Case Illustrating Treatment.[1]

By W. L. WALLACE, M. D., Syracuse.

CYSTITIS is said to be much more frequent in men than in women. I am inclined to believe that this idea originated in the day when ammoniacal urine was viewed as the sign and proof of a cystitis. It is now recognised, however, that in only a definite class of the cases of cystitis do we find ammoniacal urine, that is, those with residual urine. As obstruction to outflow is the chief cause of residual urine, and as residual urine soon becomes infected and therefore ammoniacal, and as men suffer more frequently from obstruction on account of the length of the urethra, liability to stricture, and enlarged prostate, it is

---

1. Read at the 36th annual meeting of the Medical Association of Central New York, held at Auburn, September 22, 1903.

not strange that we find the variety of cystitis having ammoniacal urine much more frequent in men than in women.

When, however, it is understood that cystitis is an inflammation of the bladder and not a condition of the urine, it must be allowed to be extremely common in women, and certainly is very distressing and obstinate.

### CAUSE.

The cause of every cystitis is infection, and almost any one or more of the pyogenic bacteria seem to be capable of producing an inflammation of the bladder. Cystitis ought to be divided into two general classes; first, cystitis caused by infection with germs capable of successfully attacking a healthy bladder; second, cystitis caused by an infection with germs incapable of successfully attacking a healthy bladder. Certain germs, as gonococci, will infect a healthy bladder.

On the other hand, a healthy bladder is invulnerable against most infections. It is able, for example, to contain urine loaded with bacteria excreted by the kidneys in infectious diseases; and ordinary pus germs may be injected per urethram into a healthy bladder almost with impunity. Infection alone will not produce a cystitis, unless with specific bladder germs, as gonococci. Furthermore, an injury will not produce cystitis unless infection supervenes. When we cut into a bladder, intentionally or unintentionally, as in hysterectomy for cancer of the uterus, we trim out any of the wall of the bladder that looks suspicious and then sew up the opening, leaving a catheter in the urethra, as a rule, and it heals immediately by first intention and without a cystitis. And we expect it to heal without a cystitis, knowing that if it does become infected, it will not heal.

An infection is always the cause of a cystitis but, as a rule, the infection is implanted upon some abnormal bladder condition. This condition of the bladder allows and invites infection, and the infection will continue (and recur if cured) until the bladder is normal. A calculus does not produce a cystitis, but if there is a stone present to injure the mucous membrane and prevent the bladder from completely emptying itself, the necessary germs will soon plant themselves in this soil, which has thus been prepared for their growth. Then, while it is extremely important to recognise that infection is the cause of every cystitis, in actual cases, those that we are trying to cure, we must go behind the cause, and search out the conditions that allow the germs to enter and to continue to thrive. We make cultures and easily find the cause, we call it by name and know all about it, as the bacillus coli communis, but where did it come from? Why did it act? What is the condition that allows the cystitis to continue and prevents our curing it?

We have the bacillus coli communis, but have we a stone or a tumor, or a neighboring pus accumulation, or what is the occasion of the cystitis? Furthermore, a bladder, the seat of a long cystitis, becomes damaged by the cystitis itself, so that, even if the original trouble (as a stone or a pus tube) is removed, the crippled bladder cannot now empty itself and the cystitis will persist. In these cases, heroic measures may be required to drain the bladder and put it at rest until it can recover.

Case.—History: Mrs. W., aged 48. Child, 16 years. No miscarriage. First seen September 18, 1902. Complaint: inflammation of the bladder. The cystitis was chronic with frequent acute attacks and was very distressing.

Examination: the patient weighed 200 pounds, was fleshy; rather short of breath. Heart normal. Perineum was badly torn and broken down, vulva red and open, exposing a large red meatus urinaris and a bulging anterior vaginal wall.

Urine, (several examinations): neutral to strongly acid,—1015-1025,—cloudy to clear, no albumin or sugar, bacillus coli communis in abundance, almost pure culture.

September 20, 1902, at the hospital, the patient was given ether. A sound was passed and the bladder found free from stone. Cystoscopic examination showed absence of tumor and presence of general redness of mucous membrane. The bladder was small and the walls thick. The uterus and appendages were practically normal. It seemed plain that ruptured perineum, with pouched bladder and exposed meatus, was the source of the trouble. The perineum was therefore repaired, supporting the bladder, obliterating the cystocele and closing the vulva.

The patient was then watched and treated for three months. Drugs were tried and irrigations, and drainage by catheter. No improvement took place at any time. The cystitis persisted, with the bacillus coli communis always present. The inflammation was continuous and distressing, and every two or three weeks a very acute and agonising attack, with awful spasms, constant attempts at micturition, with blood at the end of the act, made life absolutely unbearable. Nothing could now be found wrong except the thickened tender condition of the bladder itself.

December 19, 1902, I put the bladder at rest by doing Emmet's operation of cutting the vesicovaginal septum, making the opening large enough to pass the index finger from the vagina into the bladder, and sewing the vaginal and vesical mucous membranes together to prevent closure. This operation was followed by the most acute cystitis, which required heroic doses of morphine and which lasted three days and then gradually subsided. The signs of inflammation disappeared at once and, except for the nuisance of the constant dribbling of urine, she was well; gaining strength and health rapidly.

I left the wound open three and a half months, closing it April 3, 1903. No cystitis followed the operation of closure and the patient has remained well.

### CONCLUSION.

In conclusion, I offer a few remarks about the technic of closing the large artificial vesicovaginal fistula, which remained as an oval opening about one inch long by one-half inch transversely. The books say to pare the edge and sew up, or to split the edge and sew up the bladder and vagina separately. It is certainly very difficult to split the edge and separate the bladder and the vagina, and Kelly says that to do this operation a particularly constructed knife, like a scythe, is needed. It occurred to me that it was exceedingly easy to cut the vagina from the bladder when normal, as in a cystocele operation. I, therefore, used a method which I have since tried with great advantage in repairing rectovaginal fistula.

In a cystocele operation, an elliptical incision is made through the anterior wall of the vagina down to the bladder, and the vaginal wall is dissected away, leaving the bladder exposed, perhaps two inches by one inch, more or less. Then silkworm gut stitches are passed transversely, drawing the opening in the anterior vaginal wall shut, just catching into the bladder so as not to leave a pocket. Of course, all tension is on the rigid vagina, any surplus bladder wall is folded up as a ridge in the bladder cavity, and as a fissure toward the vagina, with its two sides in broad apposition.

To close this fistula I operated exactly as if there were no fistula present, and I desired to do a cystocele operation. An elliptical incision was made about three-fourths of an inch beyond the fistula and the anterior vaginal wall easily peeled up and cut away, from the incision to the opening, leaving exposed an elliptical portion of the external surface of the bladder wall with the opening into the bladder cavity at its center. In other words, a large hole was punched through the anterior vaginal wall, overlapping the small hole which existed through the bladder wall. Now, by pulling the vaginal opening shut, the bladder opening was closed without tension, and more than closed, the external surfaces of the bladder being broadly approximated and the edges turned up into the bladder cavity. The silkworm-gut stitches passing across from vaginal wall to vaginal wall are caught into the bladder, holding this fold together. This method combines the principles of both classical operations. The edges are pared, but the external edge is pared enormously, and the rigid vagina takes all tension from the bladder, lateral incisions being made through the vagina if necessary in order to close the vaginal opening. Much time is saved in splitting the septum toward the edge of the fistula, and in using one set only of temporary sutures.

In this case the bladder was so completely closed without tension, held by the silkworm-gut stitches through the rigid vagina, that I considered it safe; and as I feared cystitis, I did not put in a self-retaining catheter. The patient did not even require catheterisation after the operation, but got out of bed and urinated; at first often (every one to one and a half hours, and then at longer intervals), and soon normally. The wound healed by first intention, and the operation is an illustration of the amount of cutting a bladder will stand without cystitis.

